# A novel bioactive peptide-peptoid hybrid of alpha-calcitonin gene-related peptide protects against pressure-overload induced heart failure

**DOI:** 10.3389/fphar.2025.1692472

**Published:** 2025-11-10

**Authors:** Ambrish Kumar, Sarah Deloach, Lernix Williams, Donald J. DiPette, Jay D. Potts

**Affiliations:** 1 Department of Cell Biology and Anatomy, School of Medicine, University of South Carolina, Columbia, SC, United States; 2 Department of Biomedical Engineering, College of Engineering and Computing, University of South Carolina, Columbia, SC, United States; 3 Department of Internal Medicine, School of Medicine, University of South Carolina, Columbia, SC, United States

**Keywords:** cardioprotection, cardiovascular diseases, CGRP agonist analog, heart failure, peptide-peptoid hybrid, therapeutic agent, vasodilator, NMEG

## Abstract

**Background:**

Alpha-calcitonin gene-related peptide (α-CGRP) is a cardioprotective neuropeptide. However, due to low bioavailability, its use as a therapeutic agent is limited. The aim of the present study was to develop a stable and bioactive α-CGRP analog and to determine its cardioprotective effects in a mouse model of heart failure (HF).

**Methods:**

We chemically synthesized a peptide-peptoid hybrid: human α-CGRP containing two monomers of N-methoxy-ethyl glycine peptoid at the N-terminus (NMEG-CGRP). The toxicity, bioactivity, and stability of NMEG-CGRP were determined by MTT-cell viability assay, mouse blood pressure measurement, and *in-vitro* digestion with Insulin-degrading enzyme (IDE) followed by LC-MS, respectively. Male C57BL6 mice were underwent transverse aortic constriction (TAC) and were divided into: Sham, Sham+NMEG-CGRP, TAC, and TAC+NMEG-CGRP. Two-day post-TAC, NMEG-CGRP (3.6 mg/kg/mouse) was administered subcutaneously on alternate days, for a total of 28 days. Cardiac function was measured weekly using echocardiography. At the endpoint, mice were euthanized, and hearts were collected for analysis.

**Results:**

Our results demonstrated that NMEG-CGRP was non-toxic to rat H9C2 cells, more stable to IDE digestion, and bioactive. TAC-induced pressure-overload decreased ejection fraction and increased cardiac hypertrophy and dilation, fibrosis, apoptosis, oxidative stress, and macrophage infiltration in the left ventricles. NMEG-CGRP administration significantly attenuated these TAC-induced adverse cardiac effects in the HF mice.

**Conclusion:**

Together, our results demonstrated that NMEG-CGRP is a non-toxic, stable, and bioactive CGRP-analog, and protects against pressure-induced HF in mice. Thus, NMEG-CGRP is a promising novel CGRP-analog that may be used in the treatment of HF and potentially other cardiac diseases.

## Introduction

Alpha-calcitonin gene-related peptide (α-CGRP) is a 37-amino acid regulatory neuropeptide and a potent vasodilator having positive chronotropic and inotropic effects (Brain et al., 1985; Russell et al., 2014; Kumar et al., 2019a). Pharmacological studies established a cardioprotective role for α-CGRP in normal cardiovascular function and in a number of heart and vascular diseases, including heart failure (HF) (Russell et al., 2014; Kumar et al., 2019a). As a vasodilator, α-CGRP is known to lower blood pressure (BP) in normotension and hypertension (Franco-Cereceda et al., 1987; DiPette et al., 1989; Dubois-Rande et al., 1992; Skaria et al., 2019). Previously, our laboratory demonstrated, in α-CGRP knockout mice, that deletion of the α-CGRP gene further worsened the deleterious cardiovascular effects of transverse aortic constriction (TAC) pressure overload-induced HF in mice (Li et al., 2013). Further, we showed that exogenous delivery of native α-CGRP, via implanted osmotic mini-pump or alginate-microcapsules subcutaneously, significantly protected the heart against pressure overload-induced HF in mice (Kumar et al., 2019b; Kumar et al., 2020). Since α-CGRP has low bioavailability secondary to its rapid degradation (∼5 min in circulation), the peptide in its native form is impractical to use in cardiovascular disease treatment (MaassenVanDenBrink et al., 2016). Thus, the observed beneficial effects of α-CGRP in these studies led to the design and development of novel α-CGRP analogs with extended bioavailability on our laboratory.

**FIGURE 1:**
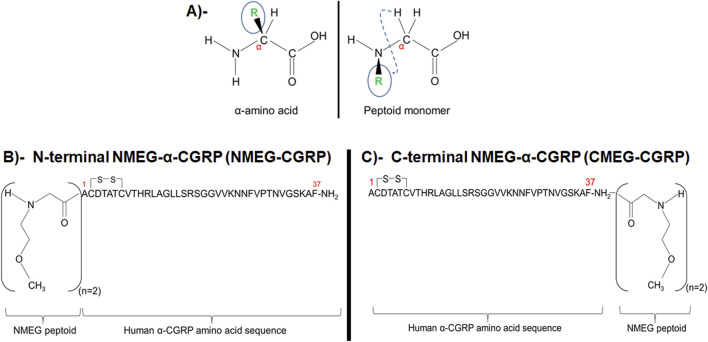
Sequence of two peptide-peptoid hybrids of NMEG-α-CGRP. **(A)** Comparison between α-amino acid and a peptoid monomer. **(B)** Human α-CGRP linked with two molecules of *N*-methoxyethylglycine (NMEG) peptoid to the N-terminal end (NMEG-CGRP). **(C)** Human α-CGRP linked with two molecules of *N*-methoxyethylglycine (NMEG) peptoid to the C-terminal end (CMEG-CGRP).

In recent years, peptoids have gained considerable attention in the modification of proteins and peptides to increase their physiological properties (Zuckermann, 2011). Peptoids are peptidomimetic molecules and a peptoid monomer is an *N*-substituted glycine molecule that is structurally identical to α-amino acid except the side chain (R-group) is attached to the nitrogen rather than the α-carbon atom (shown with dashed line in Figure 1A). The side chain (R-group) substitution makes peptoid proteolytically stable while retaining key physiochemical properties of the native amino acid (Zuckermann, 2011; Sun and Zuckermann, 2013). Peptoids are highly resistant to proteolysis, thus providing a substantial opportunity for biomedical applications. Peptoids have shown antibacterial, antifungal, and antiparasitic activities, and inhibit amyloid formation associated with Alzheimer’s disease (Uchida et al., 2009; Kapoor et al., 2011; Park et al., 2011; Bolt et al., 2018). It has been reported that coupling a peptoid *N*-methoxyethylglycine (NMEG) at the peptide C_20_ greatly improved the solubility and serum stability of the C_20_ peptide (Turner et al., 2014).

Using a peptoid chemistry approach, we chemically synthesized human α-CGRP analogs linked with two monomers of NMEG peptoid either at the N-terminal end (termed as NMEG-CGRP) or at the C-terminal end (termed as CMEG-CGRP) (Figures 1B,C). Our cell viability assay showed that both peptide-peptoid hybrids, NMEG-CGRP and CMEG-CGRP, were non-toxic to cardiac cells, however, only NMEG-CGRP was bioactive as demonstrated by a reduction in blood pressure following subcutaneous administration. Thus, we used NMEG-CGRP in the following studies to determine whether this CGRP-analog is cardioprotective in TAC-induced pressure overload HF in mice.

## Materials and methods

### Native α-CGRP and synthetic NMEG-CGRP peptide-peptoid hybrids

Human α-CGRP and mouse α-CGRP were purchased from Sigma (St Louis, MO) and GenScript (Piscataway, NJ), respectively. Human α-CGRP containing two molecules of NMEG peptoid either at the N-terminal end (NMEG-CGRP) or at the C-terminal end (CMEG-CGRP) were chemically synthesized by PolyPeptide Laboratories (San Diego, CA).

### Transverse aortic constriction (TAC) and echocardiography in mice

Eight-week-old male C57BL/6 mice, purchased from Charles River Laboratories (Wilmington, MA), were housed in the institutional animal facility on a 12 h light/12 h dark cycle with free access to food and water throughout. Animal protocols were approved by the University of South Carolina-Institutional Animal Care and Use Committee (IACUC) following the NIH guidelines.

A transverse aortic constriction (TAC) procedure was followed to induce pressure-overload HF in mice (Li et al., 2013; Kumar et al., 2019b; Kumar et al., 2020). Briefly, mice were anesthetized under 1%–2% isoflurane and the chests of these mice were opened through the suprasternal notch. A 7–0 polypropylene suture was passed under the aortic arch between the left common carotid and innominate arteries and tied around the aorta and a 27-G needle. After tying a knot, the needle was removed, and the chest was closed using a 6–0 suture. Mice were kept on the 37 °C heat pad until they recovered and buprenorphine (0.1 mg/kg/mouse) was given subcutaneously (s.c.) as post-operative care. An identical procedure except tying a knot around the aortic arch was performed in the Sham groups of mice. Two days post-TAC, mice were divided into four groups (6–7 mice/gr): i- Sham, ii- Sham+NMEG-CGRP, iii- TAC, and iv- TAC+NMEG-CGRP. A stock solution 1 mg/mL of NMEG-CGRP was prepared in sterile 0.9% NaCl soln and NMEG-CGRP at 3.6 mg/kg/mouse dose was injected s.c. in the Sham+NMEG-CGRP and TAC+NMEG-CGRP groups of mice on alternate days for a total of 28 days. At the end of the experiment, all groups of mice were anaesthetized under 4% isoflurane, when mice were fully unconscious (as checked by toe pinch), mice were decapitated using a guillotine, and hearts were collected, weighed, and photographed. Small portions of the heart were snap-frozen in the liquid nitrogen as well as fixed in 4% paraformaldehyde/PBS (pH 7.4). Mouse serum was collected and stored at −80 °C.

The short axis 2D echocardiography was performed before TAC procedure (day 0) and then weekly after start of NMEG-CGRP administration in all groups of mice under 1%–2% isoflurane anesthesia using Vevo 3100 High-Resolution Imaging System (VisualSonics Inc., Toronto, Canada) and cardiac parameters were calculated using VisualSonics Measurement Software (Li et al., 2013; Kumar et al., 2020).

### Blood pressure measurements

The mouse blood pressure (BP) was measured using a tail-cuff MC4000 BP Analysis System (Hatteras Instruments, Cary, NC). Before taking the baseline BP, mice were trained for two to three consecutive days to reduce stress-induced changes. Mice were kept on the instrument platform for at least 5–10 min to bring animal body temperature to the instrument temperature. After taking the baseline BP (0 h), a bolus dose of CGRP-analog (NMEG-CGRP or CMEG-CGRP) was injected s.c. and mice BP was recorded at various time points.

### Histochemistry

Paraformaldehyde-fixed and paraffin-embedded heart tissues were sectioned into 5 μm thick sections and immobilized on the glass slides. Heart sections were deparaffinized with xylene, rehydrated gradually with 100%, 95% and 70% ethanol, and performed Texas Red-X conjugated wheat germ agglutinin staining (WGA; Invitrogen, Carlsbad, CA) to measure cardiomyocyte cross-sectional area, Masson’s trichrome-collagen staining (PolyScientific, Bay Shore, NY) to measure fibrosis, and TUNEL staining (DeadEnd fluorometric TUNEL kit; Promega, Madison, WI) using the vendors’ protocols. The area of cardiomyocytes in WGA staining was measured using Cellpose 2.0 with the cyto2 model (Stringer et al., 2021). The percentage fibrosis in LVs was quantitated using the QuPath 0.5.0 program (Bankhead et al., 2017).

For immunohistochemistry, paraformaldehyde-fixed and paraffin-embedded heart sections (5 μm thick) were dehydrated and rehydrated as discussed previously and boiled in 10 mM sodium citrate buffer (pH 6.0) for 30 min. Heart sections were permeabilized in 0.2% TritonX-100/PBS for 10 min, blocked in 10% IgG-free bovine serum albumin (BSA)/PBS (Jackson ImmunoResearch Laboratories, West Grove, PA), and incubated in the primary antibodies for overnight at 4 °C. Secondary antibodies conjugated with Alexafluor-488 or Alexafluor-546 were added to detect protein signals and DAPI (4′, 6-diamidino-2-phenylindole; Sigma, St. Louis, MO) was used to detect the cell nuclei. After mounting tissue sections with antifade DABCO (1, 4-diazobicyclo-2,2,2-octane; Sigma) mounting media, sections were examined under the EVOS FL auto2 microscope (Invitrogen). Primary antibodies were cleaved caspase-3 (Cell Signaling Technology, Danvers, MA), 4-hydroxy-2-nonenal (4-HNE; Abcam Inc., Cambridge, MA), and 8-OHdG and macrophage marker MAC-387 (Santa Cruz Biotechnology, Dallas, TX). ImageJ Fiji program was used to calculate cleaved caspase-3 positive cells, TUNEL-positive cells, Mac-2 positive cells, and the integrated density of 4-HNE and 8-OHdG.

### *In vitro* cell viability assay

The rat cardiac H9C2 cells were maintained in Dulbecco’s Modified Eagle Medium (DMEM) cell culture medium (supplemented with 10% fetal bovine serum, 4.5 gm/liter D-glucose, and 1x penicillin-streptomycin antibiotic solution) and grown at 37 °C in a humidified incubator with 5% CO_2_. Cells in a 96-well plate were treated with 1 μM, 3 μM, and 10 µM concentrations of NMEG-CGRP and CMEG-CGRP and further grown for 4 days. The cell viability assay was performed using colorimetric MTT-cell proliferation kit I (Roche-Millipore Sigma, St Louis, MO) following the vendor’s protocol.

### *In vitro* peptide digestion and LC-MS analysis

For *in-vitro* peptide digestion, 100 µM peptide (human α-CGRP or NMEG-CGRP in sterile 0.9% NaCl soln) was incubated with 0.1 µg of insulin-degrading enzyme (IDE; R&D Systems, Minneapolis, MN) in an assay buffer (50 mM Tris-Cl, pH 7.5 + 1M NaCl) in a 25 µL reaction volume at 37 °C for 5 min, 30 min or 60 min. An equal volume (25 µL) of 10% formic acid (Sigma) was added. After heating the reaction at 95 °C for 5 min, desalting of the peptides was carried out using Pierce C18 tips (87782; ThermoFisher Scientific). For peptide-desalting, 30 µL of 2.5% trifluoroacetic acid (TFA) was added in a total 50 µL of the peptide-digestion reaction. Pierce C18 tips were wetted in 50% acetonitrile:water soln, equilibrated with 0.1% TFA, and peptides were passed through the C18 tips. After washing the C18 tips with 0.1% TFA in 5% acetonitrile:water soln, peptides were eluted in 10 µL of 0.1% formic acid in 75% acetonitrile: water soln and stored at −80 °C. Desalted peptide samples were submitted for Liquid Chromatography Mass Spectrometry (LC-MS) analysis to the University of South Carolina-Mass Spectrometry Center.

### Serum IL-6 level

The level of IL-6 in the serum collected upon sacrifice was quantitated using a Mouse IL-6 ELISA Kit (ELM-IL6; RayBiotech, GA) following vendor’s instructions. Briefly, ELISA wells coated with mouse anti-IL-6 antibody were incubated with mouse serum for 2.5 h at room temperature. After washing, biotinylated anti-mouse IL-6 antibody was added, and signals were detected using a HRP-streptavidin/TMB-substrate system. The optical density was measured at 450 nm in a spectrophotometer (BioRad iMark microplate reader).

### Statistical analysis

Data were shown as mean ± SEM until otherwise stated and *p* value less than 0.05 was considered significant. ns = non-significant (*p* > 0.05). Comparisons were made among the groups using a Student’s t-test and one-way ANOVA followed by a Tukey-Kramer *ad hoc* test (Microsoft Excel, and GraphPad Prism software, La Jolla, CA).

## Results

### Peptide-peptoid hybrids (NMEG-CGRP and CMEG-CGRP) are non-toxic

**FIGURE 2:**
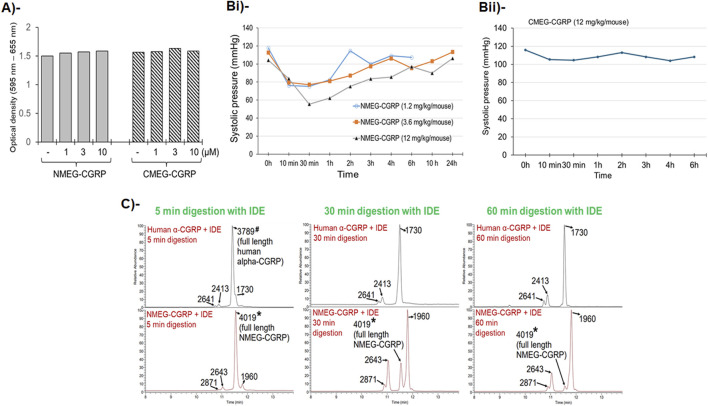
NMEG-CGRP is a bioactive and more stable analog of human α-CGRP. **(A)** Bar diagram showing MTT-cell viability assay. Rat cardiac H9C2 cells were grown in the presence of NMEG-CGRP and CMEG-CGRP for 4 days and an MTT assay was carried out. Average optical density was calculated and plotted. **(B)** Line graphs showing systolic pressure in mice. Mice were subcutaneously injected with a bolus dose of NMEG-CGRP (1.2, 3.6, and 12 mg/kg/mouse; **(Bi)** or CMEG-CGRP (12 mg/kg/mouse); **(Bii)**, and BP was measured using the mouse tail-cuff BP analysis system. **(C)** *In-vitro* digestion with insulin-degrading enzyme (IDE) followed by LC-MS analyses demonstrated that native human α-CGRP (m.wt. = 3789) completely digested by IDE within 5–30 min, however NMEG-CGRP (m.wt. = 4019) did not completely digested by IDE even after 60 min. The full length human α-CGRP is labeled with # mark. The full length NMEG-CGRP is labeled with an asterisk mark*. Peaks are labeled with peptide fragment molecular weight.

Two human α-CGRP analogs coupled with two molecules of NMEG peptoid were chemically synthesized by PolyPeptide Laboratories: 1. NMEG-CGRP (two monomers of NMEG-peptoid linked at the N-terminus of human α-CGRP; Figure 1B), and 2. CMEG-CGRP (two monomers of NMEG peptoid linked at the C-terminus of human α-CGRP; Figure 1C). Like the native α-CGRP, both peptide-peptoid hybrids contain a disulfide bond (-S-S-) at the cysteine amino acids and one -NH_2_ group (amidation) at the C-terminus. Both analogs have greater than 95% purity (as analyzed by HPLC) and are soluble in water. First, we tested if both CGRP-analogs, NMEG-CGRP and CMEG-CGRP, were non-toxic to the cardiac cells. Rat cardiac H9C2 cells were incubated with 1 μM, 3 μM, and 10 µM concentrations of NMEG-CGRP or CMEG-CGRP for 4 days and the viability of cardiac cells was determined by MTT-cell viability assay. Our results demonstrated that the measured optical density among control and treatment groups was similar (Figure 2A). These results indicated that NMEG- and CMEG-CGRP did not inhibit the growth of H9C2 cells and hence non-cytotoxic to cardiac cells.

### NMEG-CGRP is bioactive

As a potent vasodilator, α-CGRP reduces BP in animals and humans (Brain et al., 1985). To examine if the addition of NMEG-peptoid at either end of human α-CGRP retains the peptide’s vasodilator activity, NMEG- or CMEG-CGRP were injected s.c. in the wild-type mice, and mice BP was recorded using a tail-cuff BP analysis system. Our results in Figures 2Bi,Bii demonstrated that s.c. delivery of NMEG-CGRP at all given doses (1.2, 3.6, and 12 mg/kg/mouse) decreased the systolic pressure in mice in the first few hours and then returned to baseline level. In contrast, CMEG-CGRP delivery even at the highest dose tested (12 mg/kg/mouse) did not reduce systolic pressure in mice. These BP data indicated that NMEG-CGRP is a bioactive CGRP-analog, and we carried out further studies with this peptide-peptoid hybrid molecule. Since NMEG-CGRP at a dose of 3.6 mg/kg/mouse lowered blood pressure at the initial time points measured, and then returned to baseline level after 4 h, we selected this dose to determine the cardioprotective effects of NMEG-CGRP in our *in vivo* study.

### NMEG-CGRP has increased stability

Peptide α-CGRP is very short-lived in circulation and insulin-degrading enzyme (IDE) has been shown as one of the endopeptidases responsible for the degradation of α-CGRP (Kim et al., 2012). To test if NMEG-CGRP is more stable than native human α-CGRP, *in-vitro* peptide digestions were performed in the presence of IDE and generated peptide fragments were analyzed by LC-MS. The LC-MS data demonstrated that the peptide peak of full-length native human α-CGRP (m.wt. = 3789) was observed in 5 min IDE-digestion reaction but not in 30 min or 60 min IDE-digestion reaction (Figure 2C, upper panel). These spectra results indicated that native human α-CGRP was completely digested by IDE within 5–30 min. In contrast, the peptide peak of full-length NMEG-CGRP (m.wt. = 4019) was detected in all IDE-digestion reactions (5 min, 30 min, and 60 min) (Figure 2C, lower panel). This demonstrated that, even after 60 min incubation with IDE, NMEG-CGRP was not completely digested by IDE. These results suggested that NMEG-CGRP is resistant to protease degradation and more stable compared to the native human α-CGRP.

### NMEG-CGRP improves cardiac function in HF mice

**FIGURE 3:**
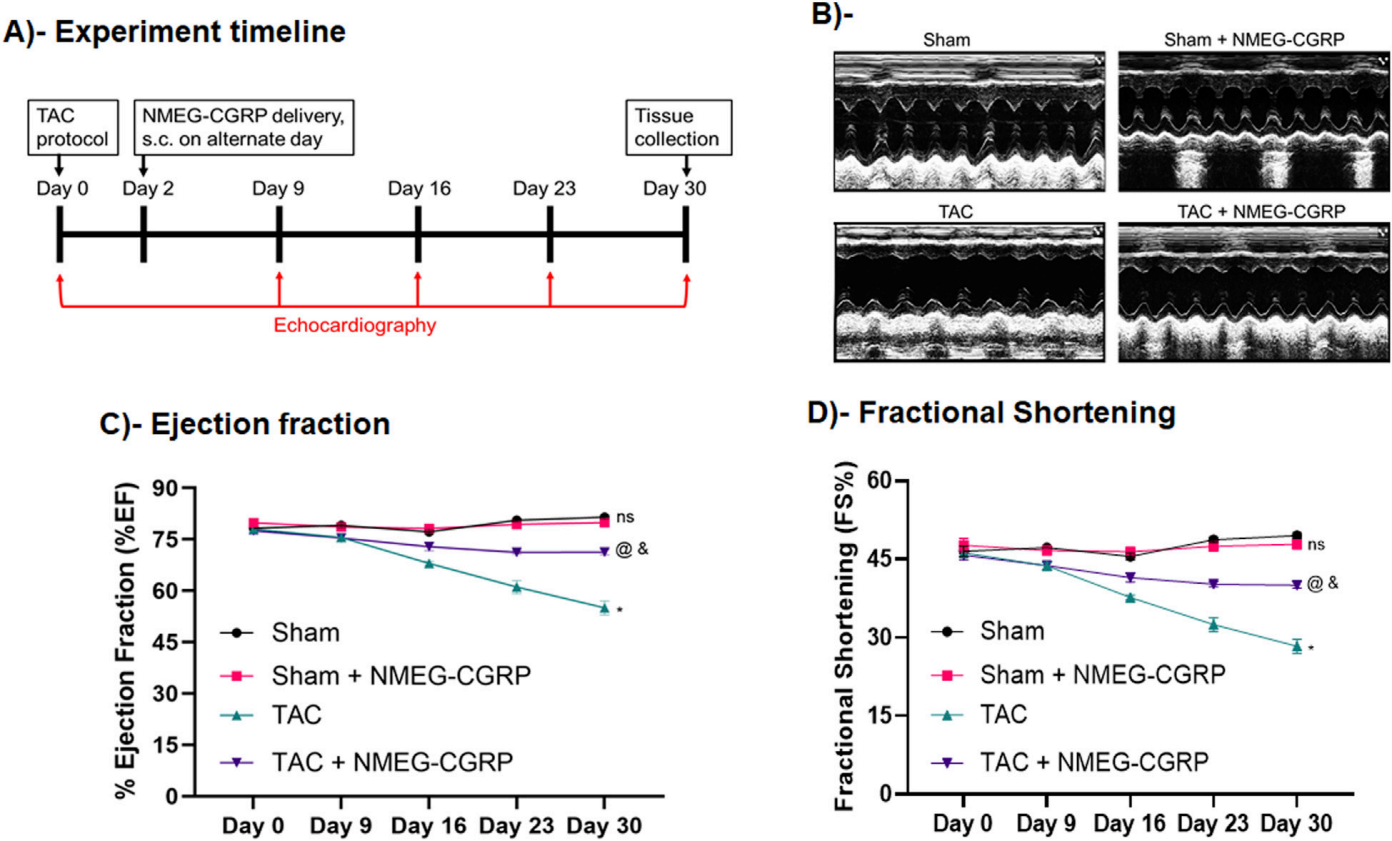
NMEG-CGRP improved cardiac function in the TAC mice. **(A)** Line diagram showing the experiment timeline. **(B)** Representative M-mode short-axis electrocardiography images taken on day 30 from Sham, Sham+NMEG-CGRP, TAC, and TAC+NMEG-CGRP group of mice. **(C)** Graph showing EF at various time points taken from Sham, Sham+NMEG-CGRP, TAC, and TAC+NMEG-CGRP group of mice. Short axis echocardiography was performed at various time points and %EF was calculated and plotted as mean ± SEM. On day 30: *Sham vs. TAC, *p* < 0.0001; ^@^TAC vs. TAC+NMEG-CGRP, *p* = 0.0008; ^&^Sham vs. TAC+NMEG-CGRP, *p* < 0.0001; ns, non-significant, Sham vs. Sham+NMEG-CGRP. **(D)** Fractional shortening (FS) from all groups of mice was plotted as mean ± SEM. On day 30: *Sham vs. TAC, *p* < 0.0001; ^@^TAC vs. TAC+NMEG-CGRP, *p* = 0.0004; ^&^Sham vs. TAC+NMEG-CGRP, *p* < 0.0001; ns, non-significant, Sham vs. Sham+NMEG-CGRP.

Our BP and peptide digestion followed by LC-MS experiments confirmed that NMEG-CGRP is a bioactive and more stable molecule. Next, we sought to determine if NMEG-CGRP is cardioprotective against HF in mice. A TAC procedure was performed to induce pressure-overload HF in mice and, after 2 days of TAC, NMEG-CGRP (dose 3.6 mg/kg/mouse) was administered s.c. in the TAC and Sham mice on the alternate days, for a total of 28 days (Figure 3A). Short-axis echocardiography was performed in all groups of mice to measure cardiac parameters (Figures 3B–D; Supplementary Figure S1). Our results demonstrated that TAC gradually reduced ejection fraction (EF) over time and, at the experimental endpoint, it was significantly less than sham mice (on day 30: sham vs*.* TAC, *p* < 0.0001). However, the administration of NMEG-CGRP significantly attenuated the reduction in EF in the TAC+NMEG-CGRP group of mice (on day 30: TAC vs*.* TAC+NMEG-CGRP, *p* = 0.0008; Figure 3C). Similarly, a reduction in fractional shortening (FS) was observed in TAC mice (on day 30: sham vs*.* TAC, *p* < 0.0001), and NMEG-CGRP administration significantly improved FS in the TAC+NMEG-CGRP group of mice (on day 30: TAC vs*.* TAC+NMEG-CGRP, *p* = 0.0004; Figure 3D). The cardiac parameters of left ventricular internal diameter at systole (LVIDs) and at diastole (LVIDd), and LV volume at systole (LV volume, s) and at diastole (LV volume, d) are shown in Supplementary Figure S1. On day 30, compared to sham, TAC increased the left ventricular internal diameter at systole (LVIDs), and NMEG-CGRP lowered the systolic internal diameter in TAC+NMEG-CGRP mice (Supplementary Figure S1). Similarly, TAC significantly increased LV volume at systole (LV volume, s), and NMEG-CGRP significantly lowered it in TAC+NMEG-CGRP mice (Supplementary Figure S1). At day 30, TAC slightly elevated LV volume at diastole (LV volume, d), and NMEG-CGRP returned it back to sham levels (Supplementary Figure S1). An increase in LVIDs as well as LV systolic and diastolic volume in TAC mice indicate that the heart is becoming dilated and is unable to contract properly likely because of a weakened heart muscle and thus unable to pump blood that leads to lower EF. These results demonstrated that NMEG-CGRP significantly attenuated the TAC-induced adverse cardiac function in the HF mice. We did not observe any abnormalities in the mice after starting NMEG-CGRP delivery in either the Sham- or TAC-mice indicating that NMEG-CGRP was not having any adverse effect on the mouse during our experimental timeline.

### NMEG-CGRP inhibits TAC-induced cardiac dilation and fibrosis in the left ventricle (LV)

**FIGURE 4:**
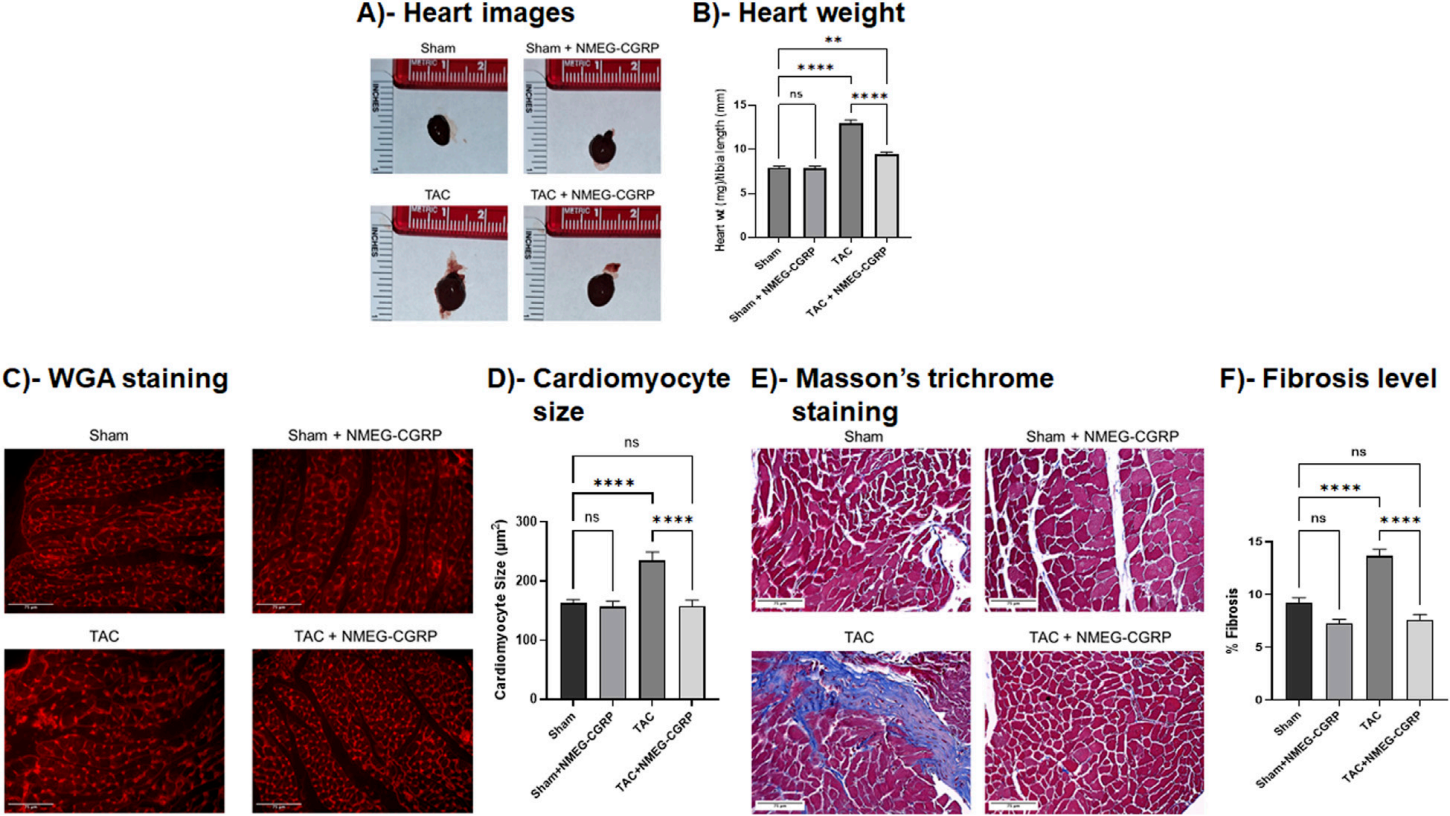
NMEG-CGRP reduced cardiac hypertrophy and fibrosis in the TAC mice. **(A)** Representative heart images from all four groups of mice. **(B)** The ratio of wet heart wt and tibia length was calculated and plotted. **p = 0.0039 and ****p < 0.0001, ns = non-significant. **(C)** Fluorescence images showing WGA staining in the LVs. Scale bar = 75 µm. The cross-sectional area of cardiomyocytes was measured and plotted **(D)**. ****p < 0.0001, ns = non-significant. **(E)** Masson’s trichrome stained images showing collagen content in the LVs. Scale bar = 75 µm. The % fibrosis was calculated and plotted **(F)**. ****p < 0.0001, ns, non-significant.

Pressure overload is known to change the heart at morphological and cellular levels (Li et al., 2013). To determine whether NMEG-CGRP administration attenuates TAC pressure-induced cellular adverse effects in the heart, heart tissue from all groups of mice was isolated, weighed, photographed, and histochemical analyses were carried out. The representative images in Figure 4A demonstrated that the gross size of hearts from the TAC mice was bigger compared to the sham mice, however the size of hearts from the TAC+NMEG-CGRP group of mice were comparable to the sham mice. The corresponding bar diagram showed that, compared to sham, the ratio of wet heart wt/tibia length was significantly higher in TAC mice (Sham vs. TAC, *p* < 0.0001) (Figure 4B). However, the wet heart wt/tibia length in TAC+NMEG-CGRP mice was significantly lower than TAC but not at the level of sham mice (TAC vs. TAC+NMEG-CGRP, *p* < 0.0001; and Sham vs. TAC+NMEG-CGRP, *p* = 0.0039). Similarly, the ratio of heart weight to body weight (HW/BW) was increased in TAC mice, and NMEG-CGRP administration significantly reduced HW/BW in TAC+NMEG-CGRP mice (Supplementary Figure S2). The area (size) of cardiomyocytes from WGA staining was measured using Cellpose 2.0 with the cyto2 model and plotted (Figures 4C,D) (Stringer et al., 2021). The representative WGA staining images and bar diagram demonstrated that cardiomyocyte size was significantly enlarged in the TAC-LVs compared to the sham-LVs (Sham vs. TAC, *p* < 0.0001), whereas NMEG-CGRP delivery significantly attenuated the increase in cardiomyocyte size in TAC-LVs (TAC vs. TAC+NMEG-CGRP, *p* < 0.0001). In addition, heart sections were stained with Masson’s Trichrome Staining to quantitate collagen content (a measurement of fibrosis) in LVs, and percent fibrosis was quantitated using QuPath 0.5.0 and plotted (Bankhead et al., 2017). The representative Trichrome Staining images of the heart and corresponding graph demonstrated that TAC significantly increased collagen content, and thus fibrosis, in the LVs (Sham vs. TAC, *p* < 0.0001). NMEG-CGRP administration significantly lowered fibrosis level (collagen content) in the LVs from the TAC+NMEG-CGRP group of mice (TAC vs. TAC+NMEG-CGRP, *p* < 0.0001) (Figures 4E,F).

### NMEG-CGRP administration attenuates increased apoptotic cell death and oxidative stress in the TAC-LVs

**FIGURE 5:**
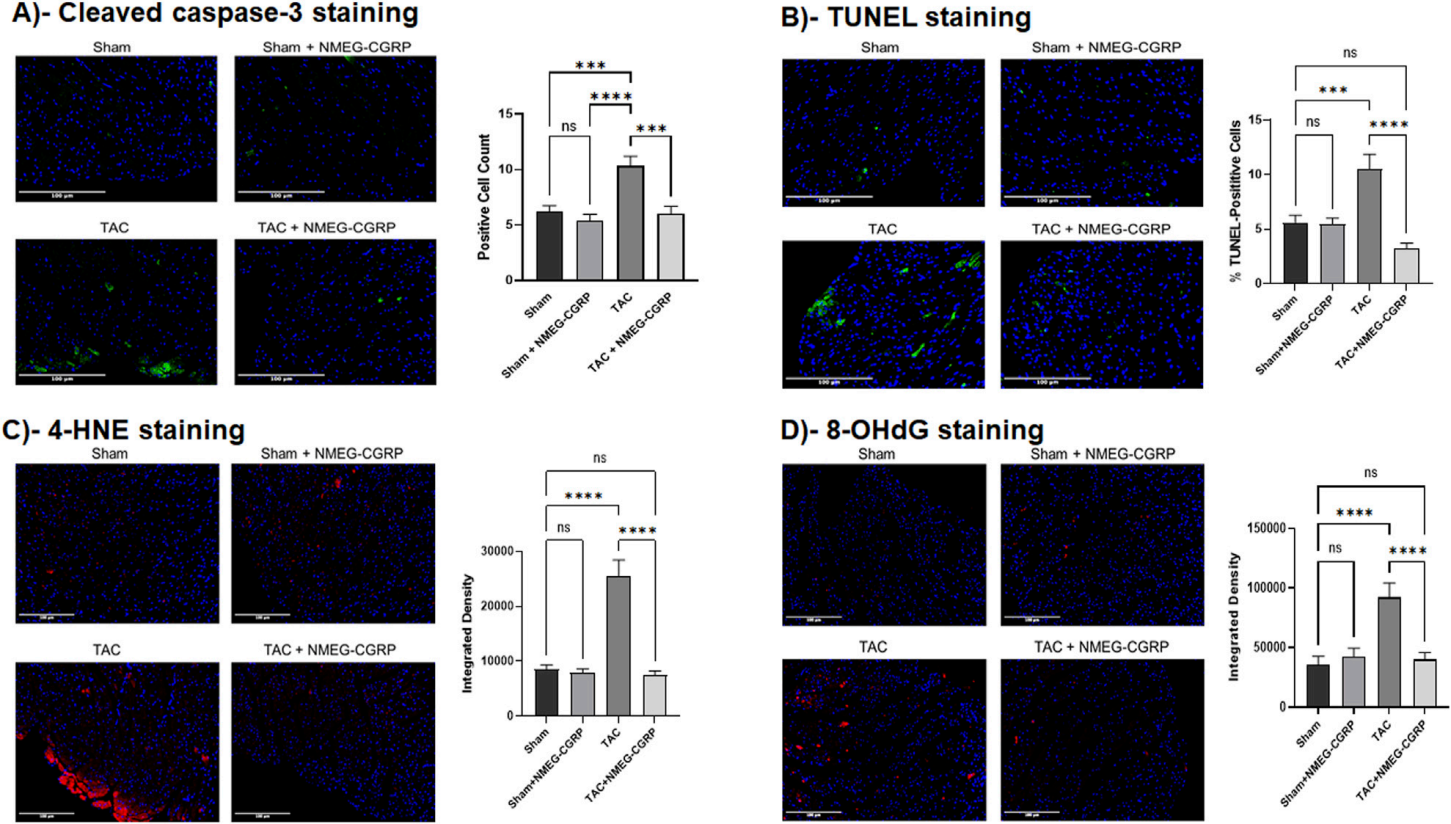
NMEG-CGRP attenuated TAC-induced apoptosis and oxidative stress in hearts. **(A)** Immunofluorescence images showing cleaved caspase-3 staining in the LVs. Cleaved caspase-3 positive cells (green) were calculated by ImageJ Fiji software and plotted. The nuclei were stained with DAPI (blue). ***p = 0.0001 and ****p < 0.0001. ns = non-significant. Scale bar = 100 µm. **(B)** Images showing TUNEL staining in the LVs. TUNEL-positive cells (green) were calculated by ImageJ Fiji software and plotted. ***p = 0.0004 and ****p < 0.0001. ns, non-significant. Scale bar = 100 µm. The nuclei were stained with DAPI (blue). **(C,D)** Heart sections were labeled with 4-HNE (red) and 8-OHdG (red) to measure oxidative stress levels in the LVs. The integrated density was calculated by ImageJ Fiji software and plotted. The nuclei were stained with DAPI (blue). ****p < 0.0001. ns, non-significant. Scale bar = 100 µm.

The level of TAC-induced apoptotic cell death in LVs was measured by performing TUNEL staining and labeling the heart tissue section with an antibody against cleaved caspase-3 (an apoptotic cell death marker). The cleaved caspase-3 images and bar diagram showed that, compared to sham, TAC LVs had an increased number of cleaved caspase-3 positive cells (Sham vs. TAC, *p* = 0.0001). Compared to TAC-LVs, the cleaved caspase-3 cell population was significantly reduced in the TAC+NMEG-CGRP LVs (TAC vs. TAC+NMEG-CGRP, *p* = 0.0001) and comparable to the sham LVs (Figure 5A and corresponding bar diagram). Similarly, the TUNEL-positive cell number increased in the TAC LVs when compared with the Sham LVs (Sham vs. TAC, *p* = 0.0004), and LVs from the TAC+NMEG-CGRP mice had significantly fewer TUNEL-positive cells compared to TAC LVs (TAC vs. TAC+NMEG-CGRP, *p* < 0.0001) (Figure 5B and corresponding bar diagram). These results demonstrated that pressure overload induced apoptotic cell death in the TAC-HF mice and NMEG-CGRP delivery significantly reduced apoptosis in the TAC-LVs.

In addition, we measured reactive oxygen species in the LVs by staining heart sections with antibodies raised against 4-HNE and 8-OHdG (Figures 5C,D). The representative 4-HNE images and bar diagram showed that, compared to Sham, TAC LVs had higher intensity for 4-HNE (Sham vs. TAC, *p* < 0.0001). However, the 4-HNE intensity was significantly reduced in the LVs isolated from the TAC+NMEG-CGRP mice and it was comparable to the Sham (TAC vs. TAC+NMEG-CGRP, *p* < 0.0001) (Figure 5C). Similarly, the integrated density of 8-OHdG was significantly higher in the TAC LVs compared to sham counterparts (Sham vs. TAC, *p* < 0.0001). The LVs from TAC+NMEG-CGRP mice had significantly lower level of 8-OHdG staining when compared with TAC LVs (TAC vs. TAC+NMEG-CGRP, *p* < 0.0001) (Figure 5D). These results demonstrated that NMEG-CGRP administrated significantly reduced TAC-induced cardiac-oxidative stress in LVs.

### Administration of NMEG-CGRP attenuates macrophage infiltration in TAC LVs and serum IL-6 level in TAC mice

**FIGURE 6:**
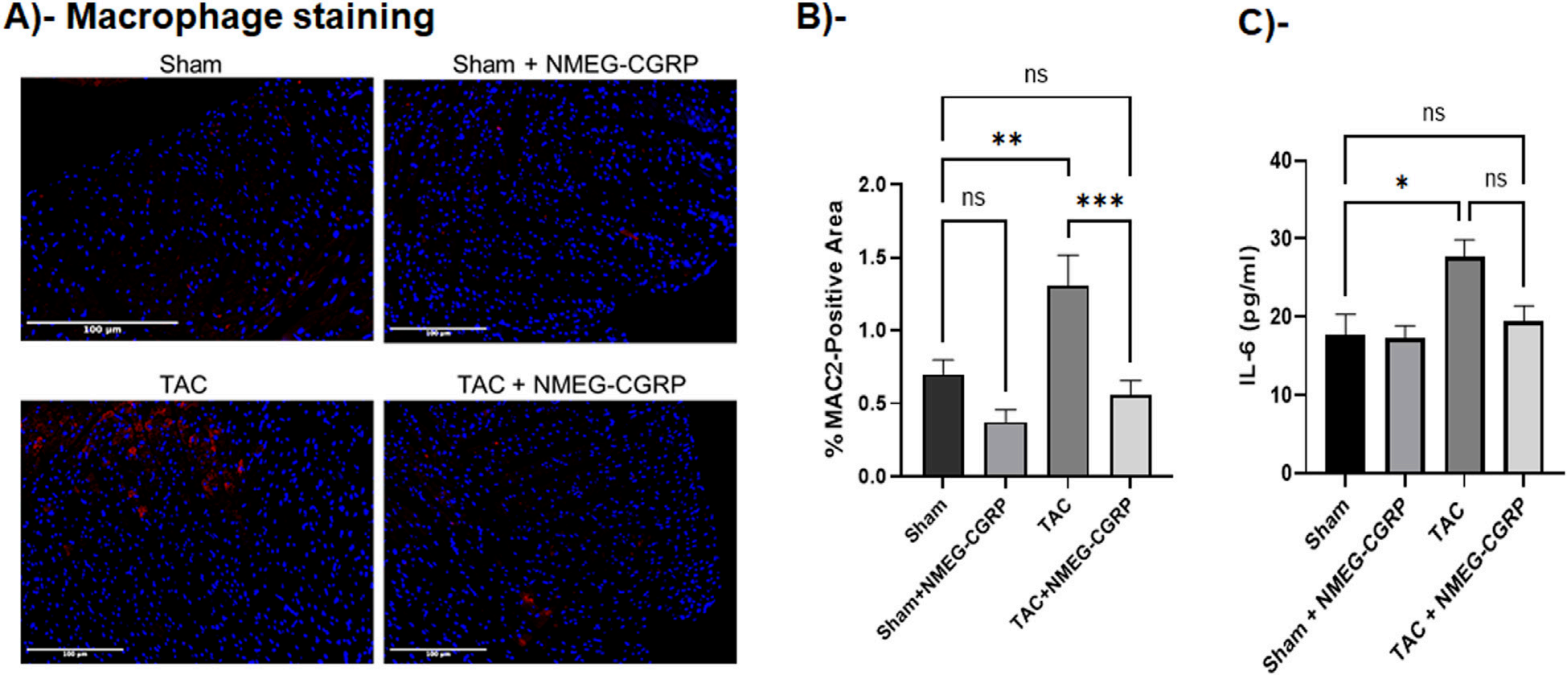
NMEG-CGRP decreased macrophage infiltration in the TAC hearts. **(A)** Representative immunofluorescence images showing the macrophage staining (red) using MAC2 antibody. The nuclei were stained with DAPI (blue). Scale bar = 100 µm. **(B)** MAC2-positive cells were quantitated by ImageJ Fiji software and plotted. **p = 0.0041 and ***p = 0.0005. ns, non-significant. **(C)** Bar diagram showing serum IL-6 level as mean ± SEM. *p < 0.05, and ns, non-significant.

It is well documented that TAC induced inflammatory responses in the hearts (Li et al., 2013). To determine if NMEG-CGRP inhibited inflammatory responses in the HF, the heart sections were labeled with an antibody specific for macrophage and macrophage-positive cells were calculated and plotted. The representative immunofluorescence images and bar graph in Figures 6A,B demonstrated that, after the TAC, the macrophage number was significantly increased in the LVs (Sham vs. TAC, *p* = 0.0041). The macrophage number was significantly reduced in the TAC+NMEG-CGRP (TAC vs. TAC+NMEG-CGRP, *p* = 0.0005) and it was to the level of sham (Sham vs. TAC+NMEG-CGRP, ns = non-significant). These results demonstrated that the TAC pressure-overload induced macrophage infiltration (a marker of increased inflammatory response) and NMEG-CGRP administration significantly reduced infiltration of macrophage (a sign of reduced inflammatory response) in the LVs from HF mice. Next, the level of the pro-inflammatory cytokine, IL-6, was measured from serum. TAC significantly increased serum IL-6 level (Sham vs. TAC, *p* < 0.05), however, NMEG-CGRP administration attenuated the elevated IL-6 level in the TAC+NMEG-CGRP mice (Figure 6C).

## Discussion

The significant findings of this study were: (a) chemically synthesized peptide-peptoid hybrid, NMEG-CGRP, is bioactive, and is non-toxic, (b) NMEG-CGRP administration attenuated the reduction in cardiac function, reduced hypertrophy and dilation, apoptotic cell death, oxidative stress, and macrophage infiltration in the LV in pressure overload-induced HF in mice.

α-CGRP is known for its vasodilatory, anti-inflammatory, and cardioprotective properties (Brain et al., 1985; Russell et al., 2014). Deletion of the α-CGRP gene makes the heart more susceptible to HF- and hypertension-induced end-organ damage (Supowit et al., 2005; Li et al., 2013). Studies from our laboratory and others showed that: 1. Infusion of α-CGRP improved myocardial contractility and cardiac function and decreased systemic arterial pressure in HF patients (Gennari et al., 1990; Dubois-Rande et al., 1992), 2. α-CGRP infusion delayed the onset of myocardial ischemia in patients suffering from stable angina pectoris (Mair et al., 1990), 3. α-CGRP delivery, via mini-osmotic pump, protected against L-NAME-induced BP increase in CGRP-KO mice (Argunhan et al., 2021), and 4. α-CGRP administration, via mini-osmotic pumps as well as alginate-microcapsules, attenuated the reduction in cardiac function and structure as well as reduced apoptosis and oxidative stress in pressure-overload induced HF in mice (Kumar et al., 2019b; Kumar et al., 2020). In addition, Aubdool et al. demonstrated that administration of an acylated form of α-CGRP significantly improved cardiac function in rodent models of Angiotensin II-induced hypertension and abdominal aortic constriction (AAC)-induced HF (Aubdool et al., 2017). Similarly, a CGRP-analog protected against experimental acute MI in rats (Bentsen et al., 2022). Together, these pharmacological studies in humans and animals established that exogenous delivery of this peptide, in its native or modified form, protected against cardiac diseases.

Using a peptoid chemistry approach, we were able to chemically synthesize two human α-CGRP analogs linked with two monomers of *N*-methoxyethyl glycine (NMEG) peptoid either at the N-terminal end (NMEG-CGRP) or at C-terminal end (CMEG-CGRP) to >95% purity. Furthermore, we showed that the addition of two NMEG-peptoid monomers at the N-terminal end of α-CGRP maintained its vasodilator property as demonstrated by the administration of a bolus subcutaneous injection of NMEG-CGRP (doses 1.2, 3.6, and 12 mg/kg body weight per mouse) lowered BP in mice. In contrast, the similar administration of CMEG-CGRP did not reduce BP. These data indicated that only NMEG-CGRP, but not CMEG-CGRP, is bioactive (Figure 2B). At the cellular level, α-CGRP interacts with its multi-complex receptor components: CLR, RAMP-2, and RCP which leads to its cellular functions (Barwell et al., 2012; Russell et al., 2014). It might be possible that NMEG-peptoid insertion at the C-terminal end altered the three-dimension structure of α-CGRP resulting in the inability of CMEG-CGRP to bind to its receptor complex and its lack of downstream signaling. However, more research is warranted to confirm this possibility.

Our LC-MS data confirmed that, compared to native CGRP, NMEG-CGRP is more biochemically stable, as it remained protected against the CGRP-degrading enzyme, IDE, for up to 1 hour, while IDE cleaved human α-CGRP within a 5–30 min time duration (Figure 2C). We further demonstrated that NMEG-CGRP was effective in ameliorating the reduction in cardiac function in pressure-overload induced HF. TAC-induced pressure overload also increased cardiac hypertrophy and left ventricular dilation and promoted apoptotic cell death, fibrosis, and oxidative stress in the LV. However, NMEG-CGRP significantly reduced all these TAC-induced adverse effects in HF mice. The level of apoptotic cell death markers (cleaved caspase-3 and TUNEL staining) and oxidative stress markers (4-HNE and 8-OHdG) was increased in the TAC-LVs, and NMEG-CGRP treatment attenuated apoptosis and oxidative stress in the TAC-LVs (Figure 5). These results indicated that NMEG-CGRP inhibited the generation of excess reactive oxygen species and cell death induced by pressure-overload. Our laboratory previously demonstrated that the TAC procedure induced apoptosis in the LV as early as in 3-day post TAC mice and was evident in later time points studied (14-day post TAC and 21-day post TAC) (Li et al., 2013). This is supported by other research groups that also demonstrated cardiac apoptosis in the 28-day post TAC mice (Yu et al., 2019; Jia et al., 2024). Previously, Li et al. (2013) demonstrated that the TAC enhanced macrophage infiltration in the LV of CGRP-KO mice compared to the TAC wild-type mice (Li et al., 2013). An increased number of macrophages in the TAC-LV in our study further confirmed that pressure-overload induced an inflammatory response in the heart, whereas NMEG-CGRP administration reduced this macrophage increase to the level seen in the sham-treated mice (Figures 6A,B). Furthermore, we observed an elevated serum level of IL-6, a pro-inflammatory cytokine, in TAC mice demonstrating pressure-overload induced inflammation (Figure 6C). A direct correlation of IL-6 level and severity of cardiac dysfunction has been reported in heart failure patients (Birner et al., 2007). Moreover, it has been shown that IL-6 infusion promotes LV hypertrophy, fibrosis, and dysfunction, and deletion of the IL-6 gene reverses these pathogeneses in animal studies (Lai et al., 2012; Kumar S. et al., 2019). In present study, a lower level of IL-6 in TAC+NMEG-CGRP mice, compared to TAC mice, suggested that NMEG-CGRP may protect hearts from pressure induced adverse effects, in part, through IL-6 mediated pathway. In addition, data suggest that multiple signaling molecules/pathways related to fibrosis, inflammation, and energy-metabolism e.g., renin-angiotensin system, catecholamines, Ca^2+^/CaM dependent protein kinase II (CaMKII) signaling, transforming growth factor- β (TGF-β) signaling via Smad2/3 and Smad1/5 pathway, NF-κB and mTOR signaling, PI3K/Akt/mTOR pathway, and STAT3-MAPK pathway, are involved in inducing cardiac hypertrophy and fibrosis during the progression of heart failure and all are regulated in some fashion by CGRP (Rockman et al., 1994; Mishra et al., 2010; Jin et al., 2018; Yu et al., 2019; Li et al., 2022; Qi et al., 2023; Gjerga et al., 2024). A role of CGRP in inhibition of renin-angiotensin system and sympathetic nervous system signaling has been reported (Oh-hashi et al., 2001; Li et al., 2004; Kumar et al., 2022). Finally, our laboratory previously demonstrated that α-CGRP administration inhibited TAC-induced activation of AMPK and sirt1 and reduced cardiac apoptosis and oxidative stress in the pressure-overload mice (Kumar et al., 2019b). Future studies to confirm the role of NMEG-CGRP in the above-mentioned pathways in providing cardioprotective effects, is warranted.

## Conclusion

Together, our study illustrates that a peptoid-based peptide modification is a novel approach to developing a new class of enzymatic biodegradable resistant and pharmacodynamically active α-CGRP analogs. This strategy ameliorates native peptide stability concerns and positions NMEG-CGRP as a novel therapeutic agent. CGRP infusion has been shown to have significant positive clinical effects in HF patients. However, due to its rapid biodegradation resulting in an extremely short half-life (approximately 5 min), the use of the native peptide form as a chronic therapeutic agent in patients with HF is extremely limited. Our state-of-the-art technology overcomes this barrier and places NMEG-CGRP as a highly effective novel therapeutic agent to treat HF and possibly other cardiovascular diseases.

## Data Availability

The raw data supporting the conclusions of this article will be made available by the authors, without undue reservation.

## Abbreviations

α-CGRP, Alpha-calcitonin gene related peptide; BP, Blood pressure; CMEG-CGRP, Human C-terminal NMEG-αCGRP; DAPI, 4′, 6-diamidino-2-phenylindole; EF, Ejection fraction; HF, Heart failure; IDE; Insulin-degrading enzyme; L-NAME, Nitro-L-arginine methyl ester; LV, Left ventricle; NMEG-CGRP, Human N-terminal NMEG-αCGRP; s.c., Subcutaneously; TAC, Transverse aortic constriction.
