# Supplementary material for: A novel bioactive peptide-peptoid hybrid of alpha-calcitonin gene-related peptide protects against pressure-overload induced heart failure
Source: Front Pharmacol. 2025 Nov 10;16:1692472. doi: 10.3389/fphar.2025.1692472 (PMC12640828; doi:10.3389/fphar.2025.1692472)
Supplement: Supplementary file 1 [file DataSheet1.docx]

**Supplementary data**

**A-**

**B-**


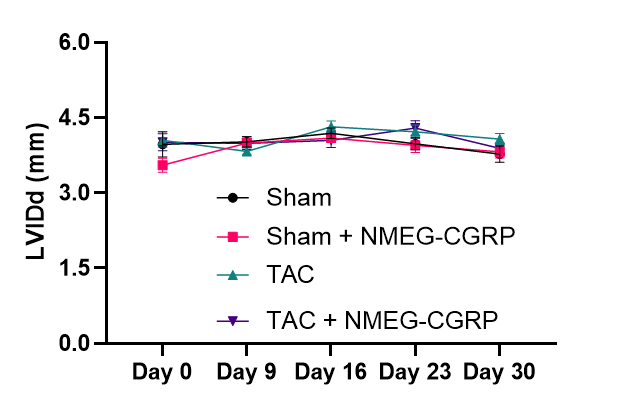

**C-**

**D-**


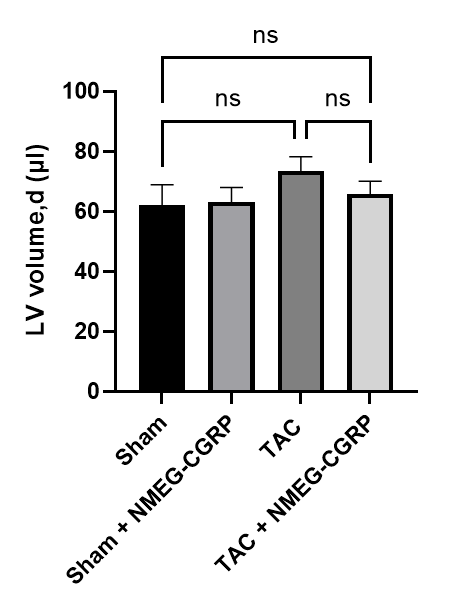


**Figure S1. Echocardiographic parameters.** Left ventricular internal diameter at systole (LVIDs; **A**) and left ventricular internal diameter at diastole (LVIDd; **B**) are shown in mean±SEM. On day 30: **p*<0.0001, sham vs TAC; ^@^*p* = 0.002, TAC vs TAC+NMEG-CGRP; ^&^*p* = 0.017, sham vs TAC+NMEG-CGRP; ns= non-significant, sham vs sham+NMEG-CGRP. **C-** Left ventricular volume at systole (LV volume, s) on day 30 post-TAC. **p* = 0.0385, sham vs TAC+NMEG-CGRP; ^***^*p* = 0.001, TAC vs TAC+NMEG-CGRP; ^****^*p* <0.0001, sham vs TAC. **D-** Left ventricular volume at diastole (LV volume, d) on day 30 post-TAC. ns= non-significant,

**Figure S2. Heart wt to body wt ratio.** The heart wt/body wt was calculated and plotted as mean±SEM. ***p* = 0.0025 and *****p* < 0.0001.
